# Landscape of targeted therapies for lung squamous cell carcinoma

**DOI:** 10.3389/fonc.2024.1467898

**Published:** 2024-10-31

**Authors:** Qiuxuan Chen, Xiaoshuo Zheng, Weiting Cheng, Jian Li

**Affiliations:** ^1^ Cancer Center, Renmin Hospital of Wuhan University, Wuhan, Hubei, China; ^2^ Institude of Experimental Immunology, University Clinic of Rheinische Friedrich-Wihelms-University, Bonn, Germany

**Keywords:** lung squamous cell carcinoma, non-small-cell lung cancer, targeted therapies, immune microenvironment, multi-target combination

## Abstract

Lung cancer, a common type of malignant neoplasm, has seen significant advancements in the treatment of lung adenocarcinoma (LUAD). However, the management of lung squamous cell carcinoma (LSCC) continues to pose challenges. Traditional treatment methods for LSCC encompass surgical resection, chemotherapy, and radiotherapy. The introduction of targeted therapy and immunotherapy has greatly benefited LSCC patients, but issues such as limited immune response rates and adverse reactions persist. Therefore, gaining a deeper comprehension of the underlying mechanisms holds immense importance. This review provides an in-depth overview of classical signaling pathways and therapeutic targets, including the PI3K signaling pathway, CDK4/6 pathway, FGFR1 pathway and EGFR pathway. Additionally, we delve into alternative signaling pathways and potential targets that could offer new therapeutic avenues for LSCC. Lastly, we summarize the latest advancements in targeted therapy combined with immune checkpoint blockade (ICB) therapy for LSCC and discuss the prospects and challenges in this field.

## 1 Introduction

Lung cancer, a leading cause of cancer-related deaths worldwide (1), is commonly classified into small-cell and non-small-cell subtypes (2). Non-small-cell lung cancer (NSCLC) accounts for approximately 85% of all lung cancer cases, with lung squamous cell carcinoma (LSCC) making up around 30% of them (3). Smoking history, prolonged exposure to harmful substances, and familial genetic factors significantly contribute to the risk of lung cancer (4–6). Patients diagnosed with lung adenocarcinoma (LUAD) often benefit more from targeted therapy and immunotherapy than those with LSCC (7). Operable mutations are rarely detected in patients with LSCC, resulting in limited treatment options (8). Currently, the primary treatments for LSCC encompass surgical resection, chemotherapy, radiotherapy, targeted therapy, and immunotherapy (6, 9, 10). The standard treatment of early and middle-stage LSCC is primarily surgical treatment, supplemented by radiotherapy and chemotherapy, and the advanced treatment is mainly chemotherapy. However, surgical resection is often unfeasible for advanced LSCC cases (11), and chemotherapy and radiotherapy can frequently lead to toxicity and drug resistance (12, 13). As an emerging approach in LSCC therapy, targeted therapy holds promise in improving patient survival rates and quality of life by inhibiting specific signaling pathways involved in cancer cell growth. Prominent pathway alterations, such as phosphoinositide 3-kinase (PI3K)/protein kinase B (AKT), epidermal growth factor receptor (EGFR), fibroblast growth factor receptor (FGFR), cell cycle protein-dependent kinases 4 and 6 (CDK4/6), and rat sarcoma (RAS) pathways, have been observed. While treatment options for the two subtypes of NSCLC, namely LSCC and LUAD, were previously similar (14), LSCC exhibits a higher prevalence of certain mutated genes, including *tumor protein p53*, *glutamate receptor, metabotropic 8*, *Erb-B2 receptor tyrosine kinase 4*, *Kelch-like ECH-associated protein 1 (KEAP1)*, and *mucin 16* (15). However, a single targeted therapeutic approach still presents certain challenges owing to drug resistance and tumor heterogeneity.

Immunotherapy treatment for LSCC shows potential benefits, particularly in histological and mutational states (16, 17). Immune checkpoints, such as programmed death receptors and their ligands, naturally regulate the immune system to prevent overreaction and autoimmune diseases (18). However, tumor cells often exploit programmed death ligands on their surface to bind to programmed death acceptors, impeding the function of T cells, thereby evading immune surveillance (19, 20). Immune checkpoint blockade (ICB) therapy stimulates the immune system to re-recognize tumor cells and increase its aggression by inhibiting the binding of tumor cells to programmed death receptors and their ligands (18, 21). Current ICB first- and second-line therapies for advanced LSCC include programmed cell death protein 1 (PD-1)/programmed cell death ligand 1 (PD-L1) and cytotoxic T-lymphocyte associated protein 4 (CTLA-4) inhibitors (22). Despite these advances, further comprehensive studies are required to address issues such as the search for novel medicines, immune tolerance management, and hazardous side effects (23, 24). ICB therapy can cause unique toxicity by enhancing immune response, consequently inducing immune-related adverse events (25). Additionally, many patients with LSCC develop immune escape owing to the human leukocyte antigen gene mutations (26). Combining immunotherapy with other therapies, including targeted therapy, may offer a reliable solution. Some classical signaling pathways, such as PI3K and CDK4/6, are associated with a tumor mutational burden (TMB) state. Novel targets such as nuclear receptor-binding SET domain protein 3 (NSD3), lysine methyltransferase 2 D (KMT2D), and ubiquitin-specific peptidase 28 (USP28), are related to tumor microenvironment (TME) and immune cells. A recent study involving 1008 patients with LUAD and LSCC suggests that identifying elevated PD-L1 expression levels could effectively guide targeted therapy. This is because PD-L1 is more prevalent in men, smokers, and squamous cell carcinoma tumors with a maximum diameter >3 cm, poorly differentiated, and/or high tumor node metastasis stage (27).

Future LSCC research will continue to focus on individualized treatment plans (28). This review summarizes the current knowledge of potential targetable gene alterations in LSCC, and considers repurposing some targets effective against other cancers, including LUAD. Considering the complex genome of patients with LSCC, we further discuss the possibility of multi-target combination therapy and combining immunotherapy with targeted therapy to improve the efficacy of LSCC treatment. We anticipate more advances in LSCC therapy, which will benefit the clinical outcomes of patients by broadening the pool of possible therapeutic targets, increasing drug selectivity, and implementing treatment approaches like targeted therapy in conjunction with immunotherapy or multi-target combination therapy.

## 2 Targeting classical targets in LSCC

**Figure 1:**
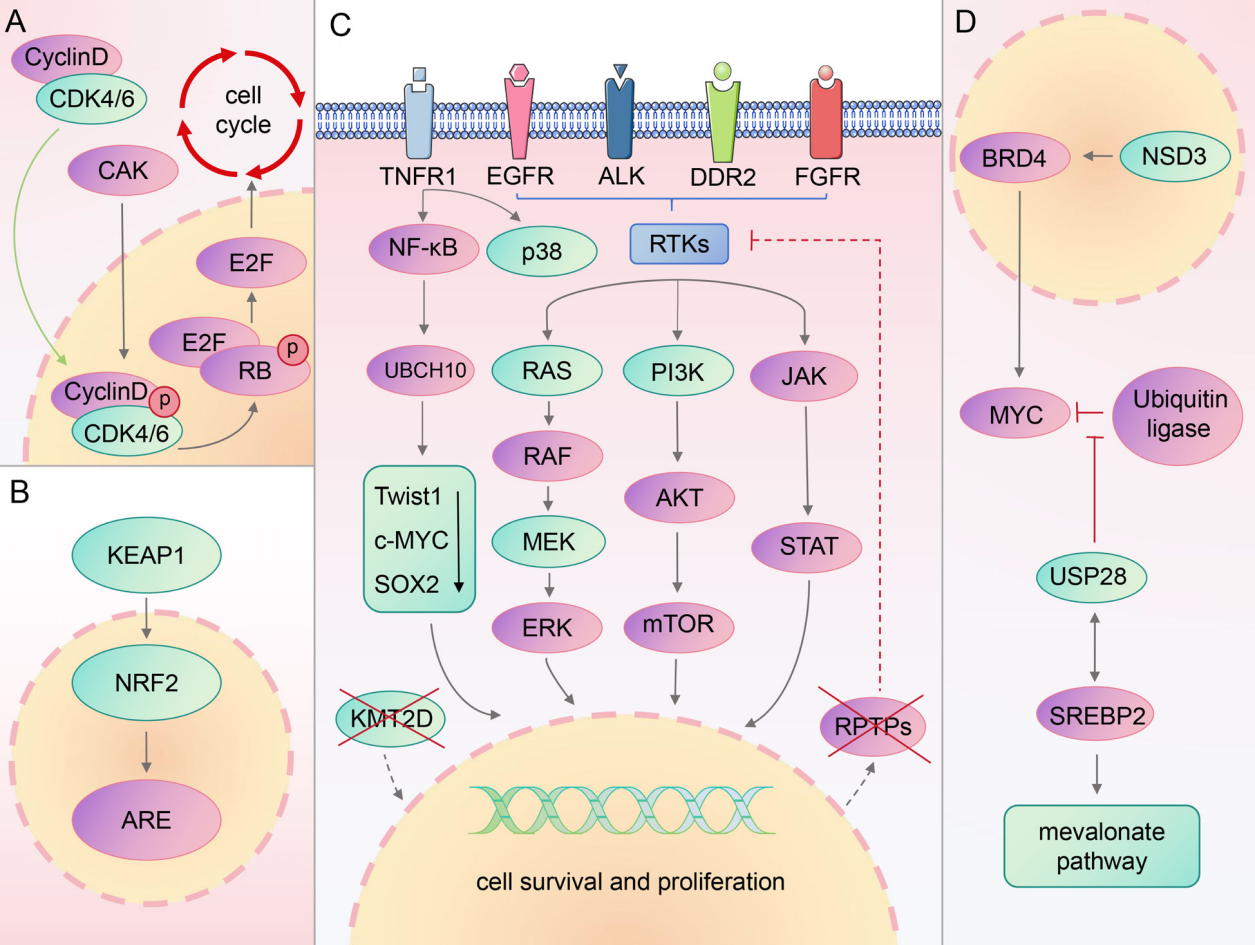
Signaling pathways associated with classical/novel targets.

Previous studies have shown that patients with LSCC exhibit numerous genetic alterations, affecting various signaling pathways. The most commonly mutated genes in lung squamous carcinoma include *phosphatase and tensin homolog (PTEN)*, *EGFR*, *cyclin dependent kinase inhibitor 2A*, and *phosphatidylinositol-4,5-bisphosphate 3-kinase catalytic subunit alpha (PIK3CA)* (29, 30). This section introduces several previously identified classical targets, along with their associated developments, paving the way for new targets and therapeutic strategies (**Figure 1**).

### 2.1 PI3K

Research has identified overactivation of the PI3K/AKT signaling pathway as a prevalent etiology among cancers (31). This pathway is disrupted in 68% of LSCC samples (32).

PI3K is divided into three main classes. Class I PI3K, which is strongly associated with cancer (33), is comprised of the catalytic subunit p110 (with four subtypes: α, β, γ, and δ) and regulatory subunit p85 (34, 35). P110α directly binds to the *RAS* gene or recruits the p85 subunit to the tyrosine phosphorylated receptor and receptor-associated adaptor proteins, signaling downstream plasma membrane-associated tyrosine kinase (36).

The primary manifestations of PI3K signal distortion in LSCC are *PIK3CA* amplification, *PIK3CA* mutation, and *PTEN* loss (37, 38). Tumor suppressor *PTEN* activation impacts the negative feedback mediating PI3K, significantly inhibiting tumor cell growth and invasion along with the activity of focal adhesion kinase (39, 40). *PIK3CA* mutations often co-mutate with other oncogenes such as *EGFR*, *RAS*, *TP53*, and so on (41), making it difficult to accurately target them with existing therapies (42). Furthermore, several clinical studies and experimental investigations have yielded negative results. When PI3K is inhibited, adaptive overexpression occurs in several compensatory pathways, leading to drug resistance (43). Furthermore, hyperglycemia has been reported in some patients treated with PI3K/AKT inhibitors (44), possibly due to the promotion of insulin-stimulated glucose uptake and storage by this pathway. Although the *PTEN*–PI3K axis has been established in LSCC studies, this pathway also plays a role in many normal cellular functions, while mutation-specific inhibitors remain unavailable. Ultimately, we need to deepen our understanding of PI3K’s function in cancer environment and enhance the durability and specificity of relevant treatments.

### 2.2 EGFR

The *erbB-1* gene encodes EGFR, a transmembrane protein crucial for cell functions such as proliferation, growth, apoptosis, repair, and survival (45, 46). Mutations in the EGFR tyrosine kinase region, mainly in exons 18–21, are strongly associated with a subset of patients with LSCC, particularly women and non-smokers (30, 47, 48). Roughly half of patients with lung cancer exhibit *EGFR* mutations, with a notable increase in EGFR protein expression in LSCC compared with LUAD (49, 50). Furthermore, the mRNA abundance of the five EGFR ligands strongly correlates with *EGFR*-amplified LSCC cohorts, but no increase in EGFR pathway activity was observed (51). This suggests that EGFR ligand abundance is a more accurate indicator of EGFR inhibitor responsiveness in these patients.

Currently, EGFR-tyrosine kinase inhibitors (TKI) are a key therapeutic option for patients with LSCC and *EGFR*-activating mutations, significantly improving clinical outcomes (28). However, owing to an altered EGFR signaling pathway and activation of abnormal bypass pathway, acquired resistance of 1^st^, 2^nd^, and 3^rd^ generation EGFR-TKI remains inevitable (52, 53). For *EGFR*-mutated patients with NSCLC who progress after EGFR-TKI treatment, a combination with immune checkpoint inhibitors (ICIs), chemotherapy, and anti-vascularization offers the greatest survival benefit, according to a statistical analysis of 2,085 patients in 17 studies (54).

### 2.3 FGFR

The FGFR family is key in tissue development and cancer progression, forming complexes with fibroblast growth factor (FGF) which activates several downstream signal transduction pathways (55, 56). Dysregulation of FGFR expression can contribute to tumor cell proliferation, survival, resistance, and immune escape (56), with LSCC showing higher FGFR1 levels than LUAD (37, 57).

FGF regulates the FGFR signaling pathway, maintaining tissue homeostasis, promoting repair and development, and influencing cell proliferation, differentiation, migration, survival, and angiogenesis (58, 59). FGF19 is crucial in prostate and breast cancer progression as it binds to the FGFR4 and Klotho complexes to initiate downstream signaling, significantly affecting LSCC development and proliferation (60–63). GLI family zinc finger 2 (GLI2), a downstream effector of FGF19, is induced by the TGF-B/SMAD pathway and is significant in promoting cancer metastasis (64). High FGF19 expression is associated with poor outcomes in LSCC, driving cell invasion through GLI2-mediated epithelial-mesenchymal transition (EMT) (65). As a result, therapeutic strategies that focus on the positive feedback loop of FGF19-GLI2 may be effective in treating LSCC.

FGFR emerges as a potential therapeutic target for LSCC (66). Current targeted therapeutics of FGFR include ortho-binding inhibitors, allosteric inhibitors, ligand traps, and small-molecule kinase inhibitors (67). *FGFR1* amplification, the most common FGFR mutation affecting approximately 20% of patients with LSCC (51), has not been proven to be a reliable predictor in therapeutic trials (57, 68). Furthermore, the complex action mechanism of NSCLC and the uncertain origins of *FGFR1* mutations warrant further research to understand or determine FGFR1 signaling in LSCC pathophysiology.

### 2.4 ALK

Anaplastic lymphoma kinase (ALK) regulates the function of the frontal cortex and hippocampus of the adult brain as well as influences tumor cell cycle control, thus impacting tumor transformation (69). Although unessential for normal growth and development, ALK's activation of signaling pathways, such as Janus kinase/signal transducer of activation, mitogen-activated protein kinase (MAPK), PI3K/AKT, and mitogen-activated protein kinase kinase (MEK) 5-extracellular signal-regulated kinase (ERK) 5, underscores its essential role in cell proliferation, differentiation, and apoptosis inhibition, making it a potential target for cancer therapy (70).

*ALK* rearrangements, particularly its fusion with *echinodermal microtubule-associated protein-like 4*, are common in NSCLC, with *ALK* mutations present in approximately 5% of cases, mostly patients with LUAD (71–74). Despite this, the large NSCLC patient population keeps ALK as a significant therapeutic target. Targeted therapy with TKIs, including crizotinib (first-generation), alectinib, ceritinib, ansatinib, brigatinib (second-generation), and loratinib (third-generation), has been developed for *ALK*-rearranged NSCLC (75–77). However, challenges including drug resistance and toxicity require further research and the development of new treatments (78).

### 2.5 RAS

When the RAS proteins are activated, they initiate several downstream signaling pathways, such as the PI3K/AKT/mTOR network, RAF/MEK/ERK pathway, and RalEGF/Ral route (79, 80). The dysregulation of the RAF/MEK/ERK signaling pathway is linked to tumor growth (81). The PI3K/AKT/mTOR pathway is associated with tumor pathologies (82). Despite initial challenges of “undruggable”, targeted allele-specific inhibitors of RAS, such as Kirsten rat sarcoma virus (KRAS)-G12C targeting drugs, have been developed, potentially changing the treatment landscape for RAS-driven tumors (83).

*KRAS* mutations are a frequent genetic cause of NSCLC, especially LUAD, but are rare in LSCC, with *KRAS* mutation rates reported between 1 and 7% in LSCC (84). Controversies exist regarding the presence of *KRAS* mutations in LSCC; some data suggest that *KRAS* mutations may be misclassified as LUAD or adenosquamous carcinoma. However, the persistence of *KRAS* mutations suggests that *KRAS* mutant LSCC is likely to exist, and future evidence may provide further clarification (85).

### 2.6 MEK

MEK is an important downstream component of the RAS with two main isoforms: MEK1 and MEK2 (86). It functions by specifically phosphorylating tyrosine and threonine residues in the activation loops of ERK1/2, playing a role in the RAS/RAF/MEK/ERK pathway (87). The *MEK* mutations are less common in patients (88). However, due to the importance of the RAF/MEK/ERK pathway, MEK is considered a potential target for new cancer therapies. For LSCC patients with *V-raf murine sarcoma viral oncogene homolog B (BRAF)* or *KRAS* mutations, monotherapy or combination therapy with MEK inhibitors, including trametinib, binimetinib, selumetinib, and cobimetinib, approved by the United States Food and Drug Administration (FDA), may be a promising treatment strategy. Compared with MEK inhibitor monotherapy, MEK inhibitor combination therapy demonstrates better therapeutic efficacy and less toxic side effects. The clinical efficacy requires further validation (89–91).

### 2.7 CDK4/6

CDK4/6 are key mediators for cell cycle transition from the G1 to the S phase (92). They contribute to the CDK-activated kinase complex phosphorylation of a CDK4/6 complex and activate cyclin D (93). Then, it phosphorylates the retinoblastoma, preventing it from repressing the E2F family of transcription factors, thus controlling the cell cycle (94). In LSCC, the cell cycle is disrupted owing to a high rate of gene inactivation that regulates CDK4/6 expression, making them potential targets for cancer therapy (95).

CDK4/6 inhibitors have shown promise in treating certain cancers, and their efficacy in treating LSCC is being investigated (96). Regretfully, monotherapy with CDK4/6 inhibitors such as palbociclib and abemaciclib has not been very successful (97, 98). Ongoing research and clinical trials are exploring the potential benefits of CDK4/6 inhibitors, both as monotherapy and in combination with other treatments (99).

### 2.8 DDR2

Discoidin domain receptor 2 (DDR2) is a member of the RTK family that plays a key role in cell proliferation and survival through EMT (100, 101). Evidence shows that DDR2 signals are closely related to the activation of PI3K/AKT and RAS/MEK/ERK pathways (102). The mutation rate of *DDR2* varies among patients with LSCC, ranging from 0 to 4.6%, possibly because of ethnic differences (103–106). A study by Miao et al. demonstrated that DDR2 mRNA levels in LSCC tissue were significantly reduced compared with normal lung tissue (105).

Dasatinib is one of the most effective drugs for inhibiting the proliferation of *DDR2*-mutated cancer cells and has been approved for leukemia treatment (107). However, its clinical application is limited because of its significant toxicity and complexity of DDR2 signal transduction in lung cancer (102, 108). In addition, the gatekeeper T654I mutation acquired on DDR2 and the deletion of *neurofibromin 1* expression through a splice site mutation can result in dasatinib resistance (109). Therefore, it is necessary to fully understand the signal transduction mechanism of DDR2 and develop the second generation of DDR2 inhibitors as soon as possible (110).

## 3 Other potential treatment targets

In addition to the conventional objectives discussed before, recent novel clinical trials have introduced innovative approaches, generating a treasure trove of valuable data for researchers. The pioneering potential targets might offer a renewed sense of optimism for LSCC treatment. This section delves into a selection of these targets.

### 3.1 NSD3

The NSD family are selective methyltransferases for histone H3 lysine 36 (H3K36) on nucleosome core particles (111, 112). This family, which operates in an auto-inhibitory state, allows H3K36 dimethylation to be catalyzed by nucleosome-based recognition and modification pathways which is crucial for maintaining chromatin stability and regulating gene expression (113).

Adjacent to *FGFR1* is *NSD3*, which regulates the histone H3 lysine 36 methyltransferase (114). The amplification of *NSD3*, strongly linked with NSD3 mRNA expression (115), is among the more common genetic alterations in LSCC. Gene set enrichment analysis revealed that MYC targets, E2F targets, G2-M checkpoints, and unfolded protein response (UPR) are highly enriched in *NSD3*-amplified tumors. Additionally, the non-inflammatory TME condition of *NSD3*-amplified LSCCs leads to a less-than-optimal clinical response (116). These findings underscore the important carcinogenic function of NSD3 in LSCC and suggest that NSD3 may influence the ability of the immune system to combat tumors.

Considering their substantial role in tumor progression (117), the NSD family is under investigation as a potential therapeutic target for various cancers. However, the development of NSD inhibitors has been slow, largely owing to limitations in bioanalysis methodologies and the unique self-inhibition ring within the SET domain of NSD, making obtaining access to its substrate binding sites challenging (118). Despite these challenges, there has been notable progress in creating small-molecule inhibitors that target specific domains of NSD3 (119, 120), largely attributed to the refinement of the molecular mechanism of histone methylation catalyzed by NSD family proteins. Several inhibitors that target the NSD3 domain or its upstream and downstream signaling targets have been reported, such as BI-9321, a selective antagonist that specifically targets the PWWP1 domain of NSD3, and BRD4, an inhibitor that targets the bromodomain and extraterminal proteins (BET) (120, 121). We look forward to further potential applications of various NSD3 inhibitors.

### 3.2 KMT2D

The KMT2 family proteins play a pivotal role in chromatin structure regulation through methylation of lysine 3 on histone H4, thereby influencing epigenetic transcriptional activation (75, 122). KMT2D is known for its role as an H3K4 mono-methyltransferase that activates enhancers (123). The *KMT2D* gene, frequently mutated in LSCC and ranking third in the mutation rate among all cancer-related genes in The Cancer Genome Atlas Pan-Cancer Atlas, is a significant factor in lymphoma and breast cancer (124–127). KMT2D functions as a tumor suppressor in ovarian malignancies and lymphoma; however, it paradoxically promotes tumor growth in gastric and breast cancers (125, 126, 128–130). This dual role highlights the complexity of KMT2D’s function in cancer biology. The role of KMT2D in LSCC remains elusive, but a recent study has shed light on its tumor suppressor function. The study discovered that KMT2D absence leads to the underexpression of receptor-like protein tyrosine phosphatases, resulting in unchecked receptor tyrosine kinases-RAS signaling. The research reveals that inhibiting Src homology-2 protein tyrosine phosphatase and pan-ERBB can mitigate receptor tyrosine kinases-RAS signaling, slow LSCC progression, and extend survival, suggesting that targeting downstream components of KMT2D may be crucial for effective LSCC therapy (131). Furthermore, KMT2D deficiency has been linked to reduced expression of the tumor suppressor gene period circadian regulator 2 in lung cancer, leading to increased glycolysis and tumors proliferating (132).

### 3.3 KEAP/NRF2

Nuclear factor erythroid 2 related factor 2 (NRF2), a basic leucine zipper (bZIP) transcription factor, is a key regulator related to cell homeostasis (133, 134). Under normal conditions, NRF2 is bound by its repressor, KEAP1, which facilitates the degradation of NRF2 by proteases, maintaining the redox equilibrium (135). The homodimerization of KEAP1 and disruption of the KEAP1-Cullin3 complex under oxidative stress diminishes KEAP1’s ability to target NRF2, allowing NRF2 to enter the nucleus and bind to the regulatory regions of antioxidant genes (136). LUAD frequently exhibits gain-of-function mutations in the *NRF2* encoding gene, whereas LSCC is characterized by loss-of-function mutations in *KEAP1*, affecting ≤21% of cases (137). Furthermore, experimental data indicates that the KEAP1/NRF2 pathway influences cell motility by inhibiting the RAS homolog family member A-Rho-associated protein kinase 1 pathway, affecting tumor cell adhesion and migration (138).

While there is currently no established cure for mutation in this pathway, many innovative and natural electrophilic compounds may serve as potential selective NRF2 activators, exhibiting anti-inflammatory properties (133). This is particularly relevant as NRF2 overactivation in cancer has been linked to chemotherapy and radiation therapy resistance, often resulting in less favorable patient outcomes, such as activating pathways that activate NRF2-mediated cisplatin resistance (137). Additionally, as reported by Sanchez-Ortega et al., reactive oxygen species (ROS) induction in wild-type NRF2/KEAP1 LSCC cells triggers iron death, and local ROS induced generation may be a novel therapeutic strategy for wild-type KEAP1/NRF2 LSCC (139). Surprisingly, Cui et al. found that NRF2 inhibition enhanced cell death and inhibited tumor growth in *EGFR*-mutated TKI-resistant non-small cell lung cancer, which may provide a new strategy to overcome resistance to EGFR-TKIs (29). Furthermore, the physiological mechanism of the KEAP1/NRF2 pathways is intricate, owing to the dual role of *NRF2* as both a proto-oncogene and tumor suppressor in cancer (140). There is an urgent need for pharmaceuticals that specifically target the KEAP1/NRF2 signaling pathway and the adoption of advanced therapeutic approaches such as genetic stratification (141). These approaches could potentially provide more effective treatment options for patients.

### 3.4 USP28

USP28, a member of the deubiquitinase family, plays a crucial role in ubiquitination by removing ubiquitin tags, thereby regulating protein stability and function (142). Studies have highlighted the importance of USP28 in controlling various cancers, including gliomas, bowel cancer, and breast cancer, suggesting its potential as a novel therapeutic target for LSCC (143–145).

F-box and WD repeat domain containing 7 (FBXW7), a crucial component of the ubiquitin ligase complex, targets several well-known oncoproteins for degradation, including c-MYC, neurogenic locus Notch homolog protein, and c-JUN (145). USP28 disrupts this process by preventing FBXW7-mediated ubiquitination (146). Experimental evidence has demonstrated that inhibiting USP28 significantly curtails tumor growth in LSCC. This therapeutic response to USP28 inhibition occurs regardless of *FBXW7* and *USP28* mutations, underscoring USP28's promise as a therapeutic target in LSCC treatment (147).

Furthermore, studies have delved into the synergistic effects of targeting both USP28 and mevalonate pathways. Abnormalities in the mevalonate pathway are closely linked to tumor growth (148). Within this context, sterol regulatory element-binding protein 2 (SREBP2), a member of the sterol regulatory element-binding proteins, is crucial for cholesterol biosynthesis and uptake (149). Studies have discovered an interaction between SREBP2 and USP28, with both co-localizing in the nucleus of LSCC cells. USP28 can modulate SREBP2 expression, thereby stabilizing it and exerting control over the mevalonate pathway, suppressing tumor growth (150). This insight paves the way for innovative LSCC therapies that target both USP28 and mevalonate pathways, offering promising avenues for treatment.

### 3.5 p38 MAPK

The p38 MAPK family plays a crucial role in the cellular stress response (151). This kinase group contributes to a stress-response pathway involving three kinase cascades that regulate cell differentiation, survival, and cycle checkpoints (152). Additionally, the dual nature of p38 MAPK has been observed in various cancers, including intestinal, liver, and breast cancer, where it influences both tumor growth and metastasis, making it a potential therapeutic target (153, 154). Despite its promise as a treatment, the clinical application of p38 MAPK inhibitors has been limited owing to systemic adverse effects. Current research is exploring combination therapy and targeting its downstream effectors to enhance its therapeutic efficacy (155). In LSCC context, the role of the p38 pathway in treatment resistance is becoming increasingly clear. Studies have shown that p38 MAPK inhibitors aid in overcoming resistance to gefitinib in NSCLC with *EGFR* mutations (156). Furthermore, the dual inhibition of FGFR and p38 MAPK has undergone a significant reduction in tumor development and proliferation (157).

### 3.6 TNFR1

Tumor necrosis factor receptor 1 (TNFR1) is a key mediator of the physiological effects of TNF (158). It activates the nuclear factor-κB (NF-κB) and MAPK pathway, causing necrotic apoptosis (159). The NF-κB pathway, connected to TNFR1, is an important molecular mechanism involved in various cellular processes such as innate immunity, inflammation, cell development, survival, and proliferation (160, 161).

TNFR1, through the NF-κB signaling pathway, stimulates the production of ubiquitin-conjugating Enzyme H10 (UBCH10), an E2 ubiquitin-conjugating enzyme. Activated UBCH10 reduces twist-related protein 1, c-MYC, and SRY-box transcription factor 2 (SOX2) levels, leading to the transformation of differentiated LSCC into dedifferentiated spindle cell carcinoma, contributing to the last stage of LSCC (162). The TNFR1–UBCH10 axis plays a crucial role in LSCC development and metastasis, positioning TNFR1 inhibitors as potential novel treatments for LSCC (**Table 1**).

**Table 1:** Existing drugs that might be used in targeting LSCC.

Drugs	Target	Drug type	Cancer type
Piqray (alpelisib)	PI3K	Small molecular inhibitor	Breast cancer
Ukoniq (umbralisib)	PI3K	Small molecular inhibitor	Lymphoma
Gedatolisib	PI3K/mTOR	Small molecular inhibitor	Colorectal cancer
Truqap (capivasertib) with fulvestrant	HR-positive and HER2-negative with PIK3CA/AKT1/PTEN-alterations	Small molecular inhibitor	Breast cancer
Anti-EGFR/VEGFR2 BsAb	EGFR and VEGFR2	Antibody	Triple-negative breast cancer
Erbitux (cetuximab)	EGFR and KRAS	Antibody	Colorectal cancer or squamous cell carcinoma of the head and neck
Futibatinib	FGFR	Small molecular inhibitor	Head and neck cancer
CYY292	FGFR1	Small molecular inhibitor	Locally advanced and metastatic cancer
Sulfatinib	FGFR1	Small molecular inhibitor	Osteosarcoma
Naporafenib	RAS	Small molecular inhibitor	Melanoma
PLX8394	RAF	Small molecular inhibitor	Solid tumor
NFX-179	MEK	Small molecular inhibitor	Cutaneous squamous cell carcinoma
Tepmetko (tepotinib)	MET	Small molecular inhibitor	Non-small-cell lung cancer
Palbociclib	CDK4/6 and STAT3	Small molecular inhibitor	Breast cancer
Thapsigargin	NSD3 (UPR)	Small molecular inhibitor	Lung squamous cell carcinoma
SHP099	KMT2D	Small molecular inhibitor	Lung squamous cell carcinoma
ML385	NRF2 and AKT	Small molecular inhibitor	Lung squamous cell carcinoma
Otilonium bromide	USP28	Small molecular inhibitor	Colorectal cancer
Vismodegib derivatives	USP28	Small molecular inhibitor	Colorectal cancer and lung squamous cell carcinoma
Resveratrol (RES)	HMMR (CD168)	Polyphenol	Lung squamous cell carcinoma
Felodipine	NFAT1	Small molecular inhibitor	Lung squamous cell carcinoma

## 4 Targeted therapy combined with immunotherapy

At present, immunotherapy stands as an important treatment option for patients with LSCC. Each of these treatments, immunotherapy, chemotherapy, and targeted therapy, faces challenges owing to the diversity of tumor phenotypes and suboptimal clinical response when used independently. Therefore, exploring the potential for combined targeted therapy and immunotherapy could significantly improve patient outcomes.

### 4.1 Classical pathway with immunotherapy

TMB is a crucial parameter for assessing immunotherapy effectiveness (163). Patients with a high TMB are more likely to benefit from ICI medications (164). High TMB (≥10/Mb) tumors in LSCC demonstrate enhanced immune cell lysis activity and enrichment of CD8^+^ T cells compared with low TMB cancers. Furthermore, research indicates that seven pathways, including the traditional PI3K/AKT and MAPK, are significantly inversely associated with TMB in LSCC (165).

Recent research suggests that PI3K inhibitors exert specific effects on TME by regulating tumor vasculature, fibroblast activity, and associated protein secretion (166). PI3K-δ and PI3K-γ signaling pathways alter the intrinsic processes of cell populations and influence tumor immune microenvironment (TIME), thereby supporting T-cell inhibition (167). Furthermore, alterations in the PI3K pathway in head and neck squamous cell carcinoma (HNSCC) have been associated with a high density of cytotoxic cells, including central memory CD8^+^ T and memory CD4^+^ T cells, which are crucial for immunotherapy and anti-tumor immunity (168).

*KRAS* mutations, prevalent in NSCLC, have been associated with increased PD-L1 expression, proliferation of tumor-infiltrating lymphocytes (TILs) proliferation, and high TMB (84, 169). Clinical trials suggest that ICIs are effective for patients with specific KRAS mutations, especially the G12C subtype (170). However, not all *KRAS* mutations are equally responsive to immunotherapy. For instance, the G12D variant is linked to lower TMB and, when co-mutated with *TP53*, shows reduced PD-L1 expression and immune infiltration (171). Given the prevalence of the G12C subtype in *KRAS* mutations and the FDA-approval of targeted drugs for this subtype, the synergistic approach of immunotherapy and KRAS targeted therapy holds considerable promise (83).

Additionally, the cell cycle pathway shows a significant positive correlation with TMB in both LUAD and LSCC. Inhibition of CDK4/6 is known to enhance T cell activation, bolster tumor immunological infiltration, reinforce immunological memory, and amplify the anti-tumor immunity elicited by anti-PD-1 antibodies (172, 173). Evidence from ovarian cancer treatment indicates that the synergistic effects of anti-PD-1 and CDK4/6 targeted therapy surpass the benefits of either treatment alone (174). Innovative combinations, such as the CDK4/6 inhibitor palbociclib and the CD73 selective inhibitor AB680, have shown efficacy in colorectal cancer (175). These findings provide hope for the potential of these combinations to increase their efficacy.

### 4.2 KEAP/NRF2 with immunotherapy

The mutation of the KEAP/NRF2 pathway is associated with a poor prognosis in patients with advanced cancer (176). In addition to being a significant targeted therapeutic option, this pathway mutation influences TIME and the selection of immunotherapy for patients with NSCLC (177). Recent clinical research has highlighted certain tumor metabolic characteristics, such as NRF2-mediated glutamine metabolism, that regulate tumor TIME (178). Glutamate and glutamine metabolism is significantly upregulated in LSCC tissues and plays a significant role in immune and stromal environmental inhibition (179). Immunotherapy for lung cancer benefits from inhibiting the glutamine metabolic pathway in several manners, including the upregulation of PD-L1 expression and reactivation of CD8^+^ T cells (177, 180). Furthermore, elevated NRF2 levels induce cell senescence. These senescent cells can be eliminated by immune effector cells recruited by NRF2-induced secretory phenotype, establishing the NRF2- NRF2-induced secretory phenotype immune surveillance axis (176).

Considering the multifaceted roles of the KEAP1/NRF2 pathway in both immunotherapy and targeted therapy, combining these two approaches may enhance clinical outcomes for patients with LSCC.

### 4.3 NSD3 with immunotherapy

Considering the significant variability of tumor immunogenicity and clinical response in patients with LSCC, precise stratification of immunotherapies is crucial. The TME of *NSD3*-amplified LSCC exhibits a non-inflammatory state with diminished activity in immune-related pathways. High UPR signaling activity may be a major modulator of the non-inflammatory TME phenotype in NSD3-amplified LSCC, as suggested by further molecular characterization (116). This suggests that NSD3 influences TIME and is linked to poorer outcomes in LSCC immunotherapy. Furthermore, NSD3 mutations impact clinical outcomes as they are related to immune cell infiltration and growth signaling in pancreatic cancer (112). While there are currently no therapeutically available NSD3 inhibitors, NSD3 could potentially serve as a biomarker for immunotherapy in combination with targeted therapy.

### 4.4 KMT2D with immunotherapy

Evidence suggests that patients with mutant *KMT2D* are likely to respond favorably to immunotherapy, as indicated by the protein's high attachment to major histocompatibility class I (181). Tumors with *KMT2D* mutations have been observed to exhibit a significantly higher TMB and improved immune infiltration in TIME (125, 182, 183). Furthermore, *KMT2D* mutations have been found to enhance immunotherapy responses in certain cancers (184). DNA damage, increased mutation burden, intron retention, and activated TE expression contribute to elevated neoantigen synthesis (185). Additionally, KMT2D has been linked to mismatch repair deficiency in prostate cancer (186). *KMT2D* mutations have been identified as potential predictive markers for melanoma and effective predictors of immune response in colorectal cancer (187, 188). The combination of immunotherapy and targeted therapy for KMT2D holds promising potential for improving treatment outcomes.

### 4.5 P38 MAPK with immunotherapy

Research indicates that 3-hydroxy-3-methylglutaryl-CoA reductase, an essential enzyme in the mevalonate pathway, regulates the production of PD-1 via p38 MAPK (189). Observations suggest that inhibiting the p38 MAPK pathway stimulates CD8+/CD4+ T cells and fosters the growth of CD8+ T cells (190, 191). A potential treatment approach could involve a temporary boost in immunity by co-blocking PD-1 and p38 MAPK (191). However, p38 MAPK activation in CD8+ T cells compromises the anti-tumor efficacy of some anti-PD-L1 therapies, indicating that a combination of anti-PD-L1 and p38 MAPK-targeted therapies may hold potential (192). Furthermore, a combination of anti-PD-1 therapy and metformin, a drug known to boost the anticancer activity of natural killer cells in a p38 MAPK-dependent manner, may be effective against metastatic melanoma (193).

### 4.6 TNFR1 with immunotherapy

TNFR1 is crucial for anti-tumor immunity, potentially predicting the immune response (194). In addition, TNFR1 is required for the TNF signaling pathway in melanoma, affecting the efficacy of anti-PD-1 therapy. After anti-PD-1 therapy, TNF insufficiency reduces TIL mortality and increases CD8+ TIL numbers, with similar effects observed in lung cancer (195). Studies suggest that TNFR1 modulates immune cell expression and enhances anti-tumor activity through NF-κB and p38 MAPK signaling cascade, regulating anti-tumor immunity, which is crucial for NSCLC development (196, 197). Furthermore, TNFR1 and interferon-γ signaling cooperate to prevent multistage carcinogenesis as a compromise in either pathway may cause T lymphocytes to promote tumor formation (198).

## 5 Multi-target combination therapy

### 5.1 FGFR1 and MAPK

Although several FGFR1 inhibitors have been tested, their clinical effectiveness is limited owing to resistance in various cancers, including lung, breast, colorectal, and melanoma (199, 200). A clinical study suggests that the TAM family of tyrosine kinases, MET, neurotrophic factor receptor pathway, and MAPK pathway are major contributors to FGFR resistance. Specific genes such as *KRAS* G13D, *HRAS*, *MAP3K8*, and *BRAF* V600E within the MAPK pathway are linked to resistance against the FGFR inhibitor BGJ398 (201). The latter three gene mutations are responsible for PI3K inhibition in breast cancer and RAF/MEK inhibition in BRAF-mutated melanoma (202, 203). BGJ398 inhibits FGFR by deactivating AKT phosphorylation and temporarily suppressing ERK phosphorylation in the NCI-H2077 cell line. However, overexpression of *neurotrophic factor receptor pathway 1*, *MET*, and *HRAS* leads to the resurgence of ERK and AKT activity, indicating an up-regulation trend in ERK activity (201). Increased MAPK signaling gene expression signaling and p38 phosphorylation are common in FGFR inhibitor-sensitive cell lines. Moreover, p38 MAPK overexpression can diminish the effectiveness of FGFR inhibition, inducing resistance (157).

Specifically, in lung cancer cell lines with amplified FGFR1, MET overexpression induces resistance to FGFR inhibitors, indicating a possible inverse correlation between these pathways (204).

### 5.2 FGFR and EGFR

Commonly used EGFR-TKIs in clinical practice include gefitinib, erlotinib, and afatinib. However, drug resistance remains a challenge (205). *KRAS* G13D and *BRAF* V600E, two MAPK family members, increase resistance to erlotinib, gefitinib, and crizotinib in NSCLC and contribute to resistance against FGFR inhibitors (201). Activation of the FGF2/FGFR1 autocrine pathway in EGFR-dependent NSCLC leads to resistance to gefitinib and osimertinib, indicating a potential connection between the FGFR and EGFR pathways (206, 207). In lung cancer cell lines with the FGF2/FGFR1 pathway, targeting this resistance mechanism with EGFR-specific TKI does not significantly increase apoptosis, indicating that FGFR inhibitors may stabilize tumor progression rather than cause tumor regression (208). Therefore, combining FGFR-specific TKIs with EGFR-specific TKIs may serve as an effective targeted therapy for LSCC, potentially delaying the development of acquired resistance in EGFR-driven NSCLC.

### 5.3 PI3K and KMT2D

Studies have demonstrated that KMT2D modulates *SOX2* expression in NSCLC through a PI3K-dependent manner, significantly impacting tumor growth. A KMT2D deficiency leads to reduced phosphoinositide-3-kinase-interacting protein 1 expression and increased phosphorylated AKT, accelerating tumor growth in NSCLC by activating the PI3K/AKT pathway and upregulating *SOX*2 expression (209). Inhibiting PI3K affects tumor growth in estrogen receptor (ER)-positive breast cancer and activates glucocorticoid-regulated kinase 1 through KMT2D, regulating ER-dependent transcription via a negative feedback loop (126, 210). Patients with ER-positive breast cancer may gain clinical benefit from the combined targeted therapy of KMT2D and PI3K, potentially increasing efficacy and preventing resistance to PI3K inhibition (211, 212). Although promising, these results are preliminary and more research is required to determine the effectiveness of KMT2D and PI3K targeted therapy in LSCC treatment. Considering KMT2D’s role in the PI3K/AKT/SOX2 axis in NSCLC, which affects tumor growth, combined targeted therapy of KMT2D and PI3K may offer new treatment possibilities for LSCC.

## 6 Discussion

This review explores the current landscape of targeted therapy for LSCC, highlighting both established and emerging targets. Targeted therapies have proven beneficial in LUAD treatment (213, 214). Patients with LSCC exhibit distinct genetic alterations from LUAD; thus, their therapeutic options remain limited (215). The scarcity of therapeutic targets, coupled with modest efficacy and prevalent adverse effects, underscores the need to expand or discover new targets. The NSD family, especially NSD3, is strongly linked to the development of several malignant cancers. The pathogenic mechanism and structure of NSD3 have been largely established. The link between LSCC pathogenesis and KMT2D deficiency has been established, paving the way for the increased development of targeted drugs. NSD3 and KMT2D are promising therapeutic targets, offering hope for the enhancement of treatment strategies for LSCC. The advent of immunotherapy has significantly improved the treatment of patients with LSCC. Nonetheless, limitations such as low immune response rates and drug resistance persist. This study delves into the potential advantages of integrating immunotherapy with targeted therapy and benefits of multi-target combination therapy are discussed.

It is vital to identify appropriate biomarkers to optimize the clinical effectiveness of targeted therapies. KEAP1 has been identified as one of the significantly mutated genes in LSCC (51). In a Japanese retrospective cohort study, approximately 13.4% of patients with LSCC carried the KEAP1 mutation (216). Blocking glutamine metabolism targeting the KEAP/NRF2 pathway is a promising therapeutic strategy. The broad-acting glutamine antagonist sirpiglenastat has been shown to induce anti-tumor efficacy (217), and has therapeutic potential in HNSCC (218). In a recent Lung-MAP next-generation sequencing analysis, David Kozono’s team found that NFE2L2, KEAP1 and PARP4 are a mutually exclusive set of gene mutations (219), indicating KEAP1 as a potential biomarker in LSCC. In addition, recent research suggests that two members of the sequence similarity family, FAM20A and FAM83A, have potential clinical applications in lung squamous cell carcinoma. A recent study based on genomic databases and analysis of clinical samples showed that FAM20A was significantly reduced in LSCC and positively correlated with immune checkpoints such as CTLA-4, leading to reduced survival (220). Moreover, Lu et al. found that FAM83A, which is overexpressed in LSCC, can promote LSCC cell growth by activating the Wnt/β-catenin signaling pathway, and is a potential biomarker (221).

Epigenetic targeted drugs have promise in treating hematological malignancies and solid tumors, with tazemetostat potentially improving outcomes in solid tumors (222, 223). Furthermore, the epigenetic landscape of LSCC has become clear; for example, the demethylation of cancer/testicular antigen is highly associated with lung cancer, with its expression correlating with the immune microenvironment of NSCLC, offering diagnostic and prognostic insights (224, 225). Promoter hypermethylation promotes the transcriptional silencing of tumor suppressor genes, facilitating LSCC diagnosis and prognosis prediction. Studies have proposed various molecular drugs targeting the epigenetic network of LSCC to revert cancer cells to normalcy, including repaglinide, procainamide, and clomipramine (226). DNA methyltransferases, histone methyltransferase enhancer of zeste homolog 2, and so on are potential targets for targeted therapies, indirectly increasing targeted therapies’ efficacy (227). Additionally, NSD3 and KMT2D, as potential new targets, are vital substances in epigenetic regulation, linking targeted and epigenetic therapy. Their roles in histone methyl transfer may pave the way for LSCC innovative treatment through the synergy of epigenetics and targeted therapies. Epigenetic therapy can reverse the resistance mechanisms of gene changes and transcriptional reprogramming (228–231). The potential of combined targeted therapy of DNA methyltransferase inhibitors and venetoclax (antiapoptotic B-cell lymphoma 2 protein inhibitor) in treating hematological malignancies was investigated, and this combination therapy was recognized as a “breakthrough therapy” by the FDA for patients with specific acute myeloid leukemia (228, 232–234). Therefore, epigenetic therapy can also complement targeted therapies by combining, significantly improving efficacy.

Furthermore, emerging antibody-drug conjugates (ADCs) offer a fresh perspective. ADCs comprise a monoclonal antibody that selectively targets specific tumor cell proteins and is linked to a cytotoxic drug known as the payload (235). As of August 2023, fifteen ADCs have been globally approved, targeting molecules such as human epidermal growth factor receptor 2, trophoblast antigen 2, nectin-4, CD22, and CD33. Among these, the ADC cetuximab saratolacan, targeting EGFR, has been approved for HNSCC (236). Early trials in NSCLC suggest potential benefits from ADCs such as trastuzumab emtansine, patritumab deruxtecan, and sacituzumab govitecan (237–239). Moreover, the bispecific delta-like canonical Notch ligand 3/CD3 IgG-like T-cell engager, which binds to both delta-like canonical Notch ligand 3 and immune cells, has inspired therapeutic drugs and antibodies to enhance treatment efficacy (240). The potential of combining targeted therapeutic drugs with antibodies to form conjugates akin to the ADCs is an area of interest for researchers. Furthermore, EGFR inhibition enhances the therapeutic effect of ADC HER3-DXd, suggesting the potential of combining targeted therapy with ADCs for more effective treatment (241).

Considering the landscape of targeted therapy for LSCC, we recommend the following clinical patient selection strategies. Age, smoking status, and overall health can inform eligibility for specific treatment options. Using genomic tests to detect mutated genes and immunohistochemical tests to detect PD-L1 levels facilitates the identification of potential therapeutic targets and development of combination treatment strategies. Finally, with patient knowledge and committee approval, patients may be encouraged to participate in clinically targeted drug trials to improve treatment effectiveness. The integration of various therapeutic options, and updating of clinical practice and research are of great significance to the improvement of therapeutic benefits.

## Glossary

ADCs: Antibody-drug conjugates
AKT: Protein kinase B
ALK: Anaplastic lymphoma kinase
BRAF: V-raf murine sarcoma viral oncogene homolog B
CDK4/6: Cell cycle protein-dependent kinases 4 and 6
DDR2: Discoidin domain receptor 2
EGFR: Epidermal growth factor receptor
ER: Estrogen receptor
FGFR: Fibroblast growth factor receptor
ICIs: Immune checkpoint inhibitors
KEAP1: Kelch-like ECH-associated protein 1
KMT2D: Lysine methyltransferase 2 D
KRAS: Kirsten rat sarcoma virus
LSCC: Lung squamous cell carcinoma
LUAD: Lung adenocarcinoma
MEK: Mitogen-activated protein kinase kinase
NF-κB: Nuclear factor-κB
NRF2: Nuclear factor erythroid 2 related factor 2
NSCLC: Non-small cell lung cancer
NSD3: Nuclear receptor-binding SET domain protein 3
P38 MAPK: P38 mitogen-activated protein kinases
PD-L1: Programmed Cell Death Ligand 1
PD-1: Programmed Cell Death Protein 1
PI3K: Phosphoinositide 3-kinase
RAS: Rat sarcoma
SOX2: SRY-box transcription factor 2
TILs: Tumor-infiltrating lymphocytes
TIME: Tumor immune microenvironment
TKI: Tyrosine kinase inhibitor
TMB: Tumor mutational burden
TME: Tumor microenvironment
TNFR1: Tumor necrosis factor receptor 1
